# Syphilis screening and treatment in pregnant women in Kinshasa, Democratic Republic of the Congo and in Lusaka, Zambia: a cross-sectional study

**DOI:** 10.12688/gatesopenres.12768.1

**Published:** 2017-12-08

**Authors:** Mabel Berrueta, Maria Luisa Cafferata, Musaku Mwenechanya, Dalau Nkamba Mukadi, Fernando Althabe, Eduardo Bergel, Luz Gibbons, Alvaro Ciganda, Karen Klein, Abigail Mwapule Tembo, Friday Habulembe Mwanakalanga, Ernest Banda, Arlette Mavila Kilonga, Paul Lusamba Dikassa, Xu Xiong, Elwyn Chomba, Antoinette K. Tshefu, Pierre Buekens

**Affiliations:** 1 Institute for Clinical Effectiveness and Health Policy (IECS), Dr. Emilio Ravignani 2024, Buenos Aires, 1414 CABA, Argentina; 2 University Teaching Hospital of Lusaka, Private Bag RW1X Ridgeway, Nationalist Road, Lusaka, Zambia; 3 Kinshasa School of Public Health, University of Kinshasa, Kinshasa, Democratic Republic of the Congo; 4 Tulane University School of Public Health and Tropical Medicine, 6823 St Charles Ave, New Orleans, LA 70118, USA

**Keywords:** Syphilis screening, syphilis seroprevalence, syphilis treatment, pregnancy

## Abstract

**Background: **Congenital syphilis is associated with perinatal deaths, preterm births and congenital malformations. Low rates of syphilis screening during pregnancy and treatment of those found seropositive have been reported in the Democratic Republic of the Congo (DRC) and Zambia. We report the rates on antenatal syphilis screening, the seroprevalence of syphilis infection, and the frequency of antibiotic treatment in pregnant women screened positive for syphilis during their attendance at antenatal care (ANC) clinics in Kinshasa, DRC and Lusaka, Zambia.

**Methods: **Women attending their first ANC were enrolled consecutively during a 9-month period in 16 and 13 ANC clinics in Kinshasa and Lusaka respectively, in the context of the baseline period of a cluster trial. Study personnel collected data on women’s characteristics, the syphilis screening practices, the test results, and the frequency of treatment, that were done under routine ANC conditions and registered in the clinic records.

**Results: **4,153 women in Kinshasa and 18,097 women in Lusaka were enrolled. The frequency of screening at the first visit was 59.7% (n= 2,479) in Kinshasa, and 27.8% (n=5,025) in Lusaka. Screening test availability varied. In the periods in which tests were available the screening rates were 92.8% in Kinshasa and 52.0% in Lusaka. The frequency of women screened seropositive was 0.4% (n=10) in Kinshasa and 2.2% (n=109) in Lusaka. Respectively, 10% (n=1) and 11.9% (n= 13) among seropositive women received treatment at the first visit.

**Conclusions: **The results of the study show that screening for syphilis in pregnancy is not universal even when supplies are available. Our ongoing trial will evaluate the impact of a behavioral intervention on changing health providers’ practices to increase screening and treatment rates when supplies are available.

## Introduction

Congenital syphilis is considered a disease of major public health importance and it is associated with stillbirths, perinatal deaths, prematurity and congenital infections ^1, 2^.

Worldwide in 2012, the distribution of maternal syphilis infections and adverse outcomes varied across regions. Progress in reducing maternal syphilis has been especially limited in Africa, the region with the greatest congenital syphilis burden representing 63% of global maternal infections (585,664) and 64% of adverse pregnancy outcomes (224,761) ^3^. Despite global incremental improvements in screening and treatment nearly 1 million pregnant women are infected with syphilis each year, and it is estimated that without treatment, 350,000 will have adverse birth outcomes ^3^. In the Democratic Republic of the Congo (DRC) and Zambia, the reported rates of maternal syphilis are 2.2% and 2.4%, respectively ^3^. In DRC, 66,617 pregnant women were estimated to have probable active syphilis in 2012, but 86% of them were not screened and only 3% were screened and treated. In Zambia, 24% of pregnant women estimated with active syphilis were screened and treated ^3^. These 2012 data were estimated based on surveillance of syphilis in pregnancy reported within the HIV Universal Access and Global AIDS Response Progress Reporting (UA/GARPR) system introduced by the World Health Organization (WHO) in 2008, in which countries are encouraged to report information on syphilis screening, seropositivity, and treatment.

It is often assumed that the main challenge to improving screening for syphilis in low-resource settings is the lack of access to supplies. However, implementation research suggests that additional interventions are needed to change the behaviors of health providers and to increase the likelihood that the supplies are used ^4^. To improve syphilis screening and treatment rates we designed a multifaceted intervention based on our previous experience ^4– 6^, and which we are currently evaluating via a randomized cluster trial (ClinicalTrials.gov:NCT02353117). This trial is ongoing, but not recruiting further participants. We seek to show that combining the provision of supplies with a multifaceted behavioral intervention is more effective than providing supplies only.

The objective of this study was to assess the baseline rate of antenatal syphilis screening, the seroprevalence of syphilis infection, and the percentage of pregnant women screened positive for syphilis who were treated at first antenatal visit, among pregnant women attending antenatal care clinics (ANC) in Kinshasa,DRC and Lusaka,Zambia. Additionally, we will explore whether the availability of supplies and women characteristics were associated with antenatal syphilis screening in this population.

## Methods

### Setting and participants

This study is an analysis of the baseline data from a facility-based, two-arm parallel cluster randomized implementation trial to evaluate a multifaceted intervention designed to improve screening and treatment for syphilis in Kinshasa and Lusaka.

For the trial, after a nine-month period of baseline data collection in 29 antenatal clinics, we assigned selected antenatal clinics to either an intervention group (“Supplies+Plus”) or a control group (“Supplies”). The intervention included: 1) opinion leaders, reminders, monitoring, and feedback; 2) point-of-care rapid tests and immediate treatment if the rapid test is positive; and 3) locally packaged treatment kits (benzathine penicillin 2.4 MIU, syringe and needle, instructions, and information on side-effects). The first primary outcome of the RCT is the frequency of women screened positive for syphilis who are treated with one dose of benzathine penicillin in the first antenatal visit of their current pregnancy. The second primary outcome is the percentage of pregnant women attending antenatal care screened for syphilis during the first antenatal visit of their current pregnancy ^7^.

Outcome data were collected in French in Kinshasa and in English in Lusaka by trained study personnel in all participating clinics. Women were recruited consecutively and all women who attended antenatal care for their first visit were invited to participate in the study and asked for their written informed consent. Women with mental or physical impairments that prevented them from giving informed consent were ineligible to participate. For all enrolled women, study personnel completed an antenatal care form with data regarding the practices and procedures performed during the first antenatal care visit, including syphilis-, proteinuria-, anemia- and HIV-testing and information about their obstetric and syphilis history. These data were obtained from the source documents that each clinic usually used to report the antenatal care practices and procedures: antenatal care book, antenatal card, Prevention of Mother to Child Transmission (PMTCT) book, laboratory book and nurses’ book. Data not available in source documents were collected from the mothers.

The syphilis screening test used at participating clinics in Kinshasa during the baseline study period was the Alere Determine ^TM ^Syphilis TP test (Alere International Ltd, UK). In Lusaka, eight clinics used only a Rapid Plasma Reagin (RPR) Card non-treponemal Test, three clinics used only a rapid immunochromatographic test SD Bioline Syphilis 3.0 (Standard Diagnostic, Inc., Yongin, Korea), and two clinics used both type of tests. In Lusaka, RPR tests were conducting within their Maternal and Child Health (MCH) departments in the clinics without labs. In Kinshasa, they did not have to send the samples anywhere else for the tests to be done.

Data were collected on paper forms and entered in each country into a secure web-based open-source data management OpenClinica system ^8^, stored into password-protected server and securely transmitted to the data coordinating centre at the Institute for Clinical Effectiveness and Health Policy (IECS), Argentina. The data entry system allowed range and consistency checks to be conducted. Cross-form edits were conducted at the data coordinating centre and resolved locally. We used double data entry to assess data keying errors of all data forms.

Additionally, during the baseline data collection period, a survey was conducted in all participating antenatal clinics to provide information about the availability of ANC services and resources for syphilis screening.

Although the outcomes of the study were only measured at first antenatal care visit, all enrolled women with a positive test for syphilis who had not received treatment during the first antenatal visit were followed up by study staff to determine whether they had received treatment until the estimated date of delivery. Staff tried to contact the mothers by phone or at the following antenatal care visits, and also revised source documents at the clinics. In addition, a list of women who were screened positive but did not receive treatment at the first visit, was sent to the country principal investigators on a monthly basis in order to be shared with local health authorities.

The baseline data collection period ran from April 1, 2015 to January 13, 2016 in the 16 selected clinics in Kinshasa and from April 13, 2015 to January 10 2016 in the 13 selected clinics of Lusaka.

### Ethics statement

The study was approved by the Tulane University Institutional Review Board, New Orleans, United States; the Ethics Committee of the Ecole de Santé Publique, Université de Kinshasa, Republique Democratique du Congo; the Ethics Committee of the University of Zambia, Lusaka, Zambia; and the Ethics Committee of the Centro de Educación Médica e Investigaciones Clínicas “Norberto Quirno”, Buenos Aires, Argentina.

### Variables

The syphilis screening rate was defined as the proportion of pregnant women who were screened for syphilis in each participating clinic at the first antenatal care visit. Among women who tested positive, we report the frequency of those that were treated at the same first visit. We also report the frequency of women who received at least one dose of antibiotics between follow up and delivery Prevalence of syphilis infection was defined as the number of women who tested positive among all who were screened.

Additional maternal-level variables collected were maternal age, birth date, marital status, highest level of education completed, residence area, parity, gestational age at the time of the first antenatal care visit, and obstetric and syphilis history.

Variables collected at the clinic-level were: type of clinic (private, public or faith-based), availability of screening tests for syphilis during the baseline period, availability of laboratory, fee for antenatal care (ANC) package (which includes ANC card as well as screening for syphilis, HIV, anemia and proteinuria), and existence of any support or assistance for syphilis/HIV programs. The existence of any support or assistance for syphilis/HIV programs was defined as any program set up in the clinic that provided capacity and/or resources for syphilis and HIV screening and treatment. Additionally, we retrieved information about the availability of resources for screening for syphilis in each day in which ANC was offered.

### Statistical analysis

A descriptive analysis of the clinics’ and women’s characteristics was performed for each country. We report frequencies and percentages for categorical variables, and means and standard deviations for continuous variables.

Individual-level variables were reported using individual women as the unit of analysis. Clinic characteristics were reported as mean of proportions and standard deviation using the clinic as the unit of analysis. Proportion for screening for syphilis was reported both using individual and clinical units of analysis. Seroprevalence and treatment rates for syphilis at first antenatal care visit were estimated and reported using women as the unit of analysis.

Among the total number of days that clinics attended patients during the baseline study period, we calculated how many days each clinic had resources for syphilis screening available and how many days they reported stock-out. Then, we calculated the syphilis screening rates in days when tests were available and days with reported stock-out.

We explored whether specific individual-level factors were associated with syphilis screening at the first antenatal visit within the subgroup of women that were attended on days when tests were available. The outcome of interest was syphilis screening, defined as having screening result which was seen and confirmed by the interviewer. The exposure variables considered were maternal age, marital status, education, obstetric and syphilis history. All pregnant women for whom we had complete data on syphilis screening were included in the analysis. We examined the association using a bivariate GEE model independently for each country. PROC GENMOD of SAS version 9.4 (SAS Institute Inc., Cary, NC) was used to obtain point estimates and standard errors and to test associations. A 2-sided p-value <0.05 was considered statistically significant. The associations were judged also from the clinical point of view. If there was a significant clinical and statistic association, we would run a multivariate analysis.

## Results

**Figure 1.**
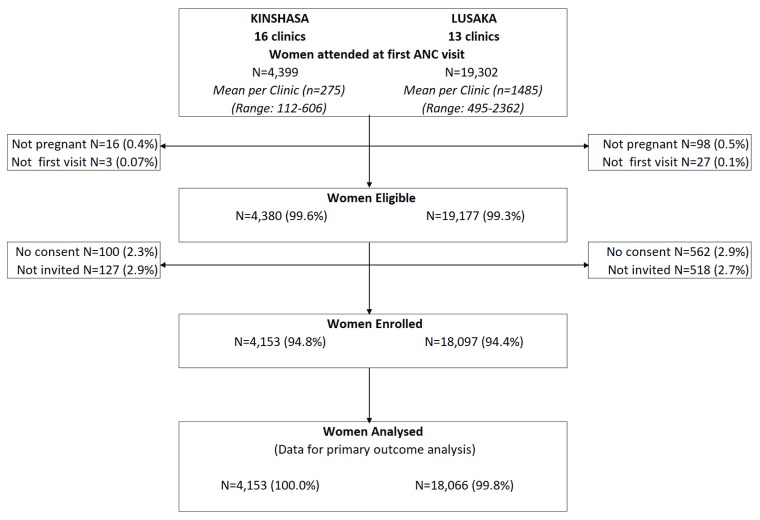
Study enrollment diagram.

Among 23,701 women attended at the first antenatal care visit 23,557 were eligible, and 22,250 were enrolled in both countries. After exclusions for missing data, the final dataset used for analysis included 22,219 women (94.3% of the initial eligible women sample); 4,153 from Kinshasa and 18,066 women from Lusaka ( Figure 1).

Table 1 shows that in Kinshasa and Lusaka respectively, of all women enrolled, 19.2% (n=797) and 14.8% (n=2,679) were less than 20 years old, 86.5% (n=3,592) and 88% (n=15,903) were married, and 62.0% (n= 2,577) and 49.3% (n=8,902) had completed primary school. In addition, 77.6% (n= 3,222) and 73.6% (n=13,255) had previous pregnancies, and 43.6% (n=1,773) and 53,6% (n=9,438) initiated ANC before 20 weeks of gestational age. Among women with previous pregnancies, 39.0% (n=1,258) in Kinshasa and 16.6% (n=2,180) in Lusaka had at least one abortion/miscarriage or stillbirth, 3.7% (n=120) and 3.9% (n=515) reported a history of preterm birth, and 6.1% (n=196) and 3.2% (n=415) a history of low birth weight infants respectively ( Table 1). Of note, 0.2% (n=6) of women in Kinshasa and 2.1% in Lusaka (n=382) recalled having had a syphilis infection in the past. ( Table 1).

**Table 1.** Characteristics of enrolled women.

CHARACTERISTICS	Kinshasa (N=4,153)	Lusaka (N=18,066)
	n/N (%)	n/N (%)
**Maternal Age**		
<20	797/4,152 (19.2%)	2,679/18,053 (14.8%)
20–34	2,788/4,152 (67.1%)	13,462/18,053 (74.6%)
> or = 35	568/4,152 (13.7%)	1,912/18,053 (10.6%)
**Residence**		
Inside the catchment area	3,409/4,153 (82.1%)	10,046/18,048 (55.7%)
Outside the catchment area	740/4,153 (17.8%)	8,000/18,048 (44.3%)
Other	5/4,153 (0.1%)	2/18,048 (0.0%)
**Education**		
Incomplete primary school or less	683/4,153 (16.4%)	4,176/18,054 (23.1%)
Completed primary school	2,577/4,153 (62.0%)	8,902/18,054 (49.3%)
Incomplete secondary school or more	893/4,153 (21.5%)	4,976/18,054 (27.6%)
**Marital status**		
Married/partner	3,592/4,153 (86.5%)	15,903/18,066 (88.0%)
Single, widowed, or divorced	561/4,153 (13.5%)	2,163/18,066 (12.0%)
***Obstetric and syphilis history***		
**Previous pregnancies**		
0	930/4,152 (22.4%)	4,763/18,018 (26.4%)
1–3	2,034/4,152 (49.0%)	10,741/18,018 (59.6%)
≥ 4	1,188/4,152 (28.6%)	2,514/18,018 (14.0%)
Previous abortions/stillbirth **	1,258/3,222 (39.0%)	2,180/13,145 (16.6%)
Previous preterm birth **	120/3,222 (3.7%)	515/13,126 (3.9%)
Previous congenital anomalies **	24/3,222 (0.7%)	44/13,119 (0.3%)
Previous low birth weight **	196/3,221 (6.1%)	415/12,878 (3.2%)
**Previous Syphilis Infection**		
Yes	6/4,153 (0.1%)	382/1,8066 (2.1%)
No	4,135/4,153 (99.6%)	17,663/1,8066 (97.6%)
Don't Know	12/4,153 (0.3%)	51/1,8066 (0.3%)
**Gestational age at first visit**		
Less or equal 20 weeks	1,773/4,064 (43.6%)	9,438/17,620 (53.6%)
More than 20 weeks	2,291/4,064 (56.4%)	8,182/17,620 (46.4%)

** Considering only those women with at least one previous pregnancy

In Kinshasa, 5 of the 16 participating clinics are public, 7 are faith-based and 4 are private. All 13 participating clinics in Lusaka are public. HIV and/or syphilis programs have been implemented in 14 of 16 clinics in Kinshasa and in all clinics in Lusaka. A laboratory for antenatal care testing is available in all Kinshasa clinics, but only in 6 in Lusaka. In Kinshasa women must pay a fee for the ANC card that allows them to access to ANC services, including syphilis testing, while in Lusaka antenatal care visits and syphilis screening are provided free of charge. ( Table 2). The baseline study period included 249 and 185 business days in which ANC care was offered at Kinshasa and Lusaka clinics respectively.

**Table 2.** Characteristics of participating clinics.

CLINICS CHARACTERISTICS	Kinshasa (n=16)	Lusaka (n=13)
	n/N (%)	n/N (%)
Public Clinics	5 (31.3%)	13 (100.0%)
Clinic with Syphilis/HIV program	14 (87.5%)	13 (100.0%)
Laboratory available	16 (100.0%)	6 (46.2%)
Fees for:		
Antenatal Care package	16 (100.0%)	0 (0.0%)
Proteinuria test	9 (56.3%)	0 (0.0%)
Syphilis treatment	0 (0.0%)	0 (0.0%)

The frequency of pregnant women who were screened for syphilis at the first ANC was 59.7% (n= 2,479) in the Kinshasa clinics, and 27.8% (n=5,025) in those in Lusaka. ( Table 3). During the 9-month study period, ANC was offered on average 122 days (SD 42) in Kinshasa clinics. The availability of supplies for syphilis screening was not constant, and varied greatly by time period and clinic. On average, supplies were available in 78 ANC days (SD 59) in Kinshasa. Similarly, the clinics in Lusaka offered ANC in 129 days (SD 43), and supplies were available in 69 ANC days (SD 38) When the frequency of screening was calculated only considering the periods in which tests were available, the figures were 92.8% and 52.0% in Kinshasa and Lusaka clinics respectively. Rates were similar among the different type of clinics in Kinshasa. Detailed data by clinic is shown in Table S1 and Table S2. Three clinics in Kinshasa did not have screening resources available at any day during the baseline period, while 5 reported never having a stock-out. All clinics in Lusaka reported stock out in part of the baseline study period. Whether the clinics in Kinshasa received support from specific programs was not associated with the syphilis screening rate ( Table S1 and Table S2).

**Table 3.** Frequencies of Syphilis Screening, Syphilis seropositivity, and treatment in seropositive women during antenatal care (ANC) in 16 clinics in Kinshasa, DRC and 13 clinics in Lusaka, Zambia.

	Kinshasa (N =4,153)	Lusaka (N =18,066)
	n/N (%)	n/N (%)
Women screened during first ANC visit	2,479/4,153 (59.7%)	5,025/18,066 (27.8%)
* -When clinics had screening resources*	2,469/2,660 (92.8%)	4,761/ 9,155 (52.0%)
Seropositive women at screening	10/2,479 (0.4%)	109/4,961 (2.2%)
Seropositive women treated at first ANC visit	1/10 (10.0%)	13/109 (11.9%)
Seropositive women treated between follow up and delivery	8/10 (80.0%)	66/109 (60.5%) *

* 25 women were unable to locate at follow up

In participating clinics, 0.4% (n=10) in Kinshasa and 2.2% (n=109) in Lusaka of women screened at first ANC visit were serologically positive ( Table 3). Among 6 women who recalled a history of previous syphilis, 2 were screened negative using a treponemal point of care test in Kinshasa. On the other hand, in Lusaka, 93/382 women with history of previous syphilis were screened and 7 of them were positive ((2/48 using a rapid treponemal test and 5/43 using a non-treponemal RPR test). (Data not shown).

The frequency of women who tested positive and received treatment at first visit was 10% (n=1) in Kinshasa and 11.9% (n=13) in Lusaka ( Table 3). Women who tested positive but were not treated at first visit were followed up to record whether they received treatment at any time between first visit and delivery. All women were followed up in Kinshasa (n=9) and 73.8% (n=71) in Lusaka. Of note, we were unable to locate 25 women after the first ANC visit in Lusaka. Treatment at any time during pregnancy was 90.0% in Kinshasa (n=9) and 72.5% (n=79) in Lusaka ( Table 3).

Table 4 shows the bivariate analysis results of the factors in association with screening rates. The absence of a partner was found to be statistically significantly associated with lower screening rates in both cities. Also, women with no previous history of abortions were associated with lower screening rates in Kinshasa only. However, the differences in screening rates among the categories of these factors were very small and not clinically relevant. As the observed differences were not clinically significant, we did not conduct a multivariate analysis.

**Table 4.** Factors associated to syphilis screening among women who were attended on days with availability of tests in Kinshasa and Lusaka.

	Kinshasa		Lusaka	
	Syphilis Screening		Syphilis Screening	
	(N=2660)		(N=9148)	
	n/N (%)	P - value	n/N (%)	P - value
***Maternal Age***				
<20	494/534 (92.5%)	0.0506	712/1341 (53.1%)	0.87812
20–34	1645/1767 (93.1%)	ref	3557/6821 (52.1%)	ref
> or = 35	330/359 (91.9%)	0.9148	491/948 (51.8%)	0.7006
***Education***				
Incomplete primary school or less	436/470 (92.8%)	0.5816	1019/2111 (48.3%)	0.8951
Completed primary school	1572/1694 (92.8%)	0.5481	2463/4536 (54.3%)	0.9793
Incomplete secondary school	461/496 (92.9%)	ref	1280/2464 (51.9%)	ref
***Marital status***				
Married/partner	2119/2276 (93.1%)	ref	4249/8065 (52.7%)	ref
Single, widowed, or divorced	350/384 (91.1%)	0.0284	513/1052 (48.8%)	0.0414
***Obstetric history***				
**Previous pregnancies**				
0	550/598 (92.0%)	0.5956	1285/2401 (53.5%)	0.4174
1-3	1221/1311 (93.1%)	ref	2825/5415 (52.2%)	ref
≥ 4	697/751 (92.8%)	0.2673	650/1288 (50.5%)	0.845
**Previous abortions/stillbirth**				
No	1698/1842 (92.1%)	0.001	4164/7935 (52.5%)	0.483
Yes	770/817 (94.2%)	ref	581/1125 (51.6%)	ref
**Previous preterm birth**				
No	2393/2578(92.8%)	0.4849	4593/8786 (52.3%)	0.0647
Yes	76/82 (92.7%)	ref	152/272 (55.9%)	ref
**Previous congenital anomalies**				
No	2453/2644 (92.7%)	*	4728/9031 (52.3%)	0.5627
Yes	16/16 (100%)	ref	13/24 (54.2%)	ref
**Previous low birth weight**				
No	2357/2535 (92.9%)	0.2942	4566/8674 (52.6%)	0.7731
Yes	112/125 (89.6%)	ref	140/268 (52.2%)	ref
**Previous Syphilis Infection**				
No	2467/2657 (92.8%)	ref	4677/8940 (52.3%)	ref
Yes	2/3 (66.7%)	0.2611	85/177 (48.0%)	0.2611
***Current pregnancy***				
**Gestational age at first visit**				
Less or equal 20 weeks	949/1023 (92.8%)	ref	2504/4732 (52.9%)	ref
More than 20 weeks	1453/1569 (92.6%)	0.2611	2126/4095 (51.9%)	0.7933

(*) *not estimable*

## Discussion

Our study found that screening for syphilis during pregnancy in ANC clinics was done in 59.7% and 27.8% of pregnant women attending the first visit in Kinshasa and Lusaka respectively. Among those who were screened, 0.4% women in Kinshasa and 2.2% women in Lusaka were found positive, and 10.0% and 11.9% of women tested positive received treatment in that first visit at each country respectively. When screening was calculated only considering days in which the clinics had availability of tests, the rates were 92.8% in Kinshasa and 52.0% in Lusaka.

This study has several strengths, including that is the baseline period of a cluster trial in two capital cities of Sub-Saharan African countries, DRC and Zambia. The study enrolled 93.8% (n=22,250) of all women attending their first ANC visit in 29 ANC clinics in Lusaka and Kinshasa during a 9-month period, supporting that the results are representative for those urban settings. The study was done under the routine ANC conditions and the screening and laboratory tests used were those currently in use in both countries, which suggests that the results reflect the reality. Finally, as this study is based on the baseline data collection period of a cluster RCT, the data collected is of high quality, according to good clinical practice standards for randomized controlled trials.

Nonetheless the study has limitations. The screening tests used were not the same in both countries (a rapid Treponemal point of care Test in Kinshasa and a non-treponemal test or rapid Treponemal Test in Lusaka), and women who were screened positive had no confirmation tests. Thus, the rates should be adjusted for fair comparisons between the two countries and with other countries, which may introduce more uncertainty. The study was conducted in the two capital cities, and the results are not generalizable either to rural areas or the country. Finally, this study was not designed to assess factors potentially associated to screening and treatment and the results should be interpreted cautiously. Moreover, this assessment was not possible for factors associated to treatment because of the limited number of positive cases.

The screening rates were not high and varied greatly between the two cities. While in Kinshasa 6 out of 10 women were screened at the first ANC visit, in Lusaka no more than 3 women in 10 received the practice. These rates are compatible with a recent analysis using model based data, which reported 27.8% and 3.3% screening rates for the whole DRC and Zambia respectively ^3^. The rates in rural areas are expected to be lower than in the capital cities ^9^. The overall screening rates at the clinics were substantially determined by the availability of the tests. Tests were available in 64% of the attending days in Kinshasa clinics and, when available, the screening rate was 92.8%. This finding suggests that the supply availability is the main barrier to screening in Kinshasa clinics. In Lusaka, the situation is somewhat different. Tests were available 49% of attending days, and the rate was almost 52% when they were available, suggesting that other important barriers to screening exist in public ANC clinics in that city. We have not found any relevant maternal factors clinically and statistically associated with screening in Lusaka. However, this study was not originally designed with that objective. The qualitative assessment of barriers that we conducted as part of the trial formative research showed that additional barriers exist in Lusaka. Barriers related to structural country constraints such as electricity power cuts, health system barriers like staff shortages, health providers’ lack of knowledge and formal training, and women’s factors such as fears of being stigmatized ^10^ were reported. Other studies in Sub-Saharan countries have reported similar findings ^11^.

The rates of syphilis infection in pregnant women were 0.4% in Kinshasa and 2.2% in Lusaka. These rates should be interpreted cautiously as they are based only on screening tests and no confirmatory tests were done. Screening using either treponemal or non-treponemal tests tend to show false positive results. A reactive, but unconfirmed, non-treponemal test may represent a biological false-positive result, whereas a reactive treponemal test alone may represent an old or previously treated infection that poses little exposure risk for the fetus ^12^. Montoya *et al.* ^13^ have reported that 60% of pregnant women with a history of old and treated infection showed positive treponemal tests results under routine conditions. When rates are estimated using these tests alone, the current recommendation is to adjust them multiplying the rate by a correction factor (0.522 for non-treponemal tests, and 0.536 factor to treponemal test) ^12^. After this adjustment, the rate of syphilis infection would be 0.2% in Kinshasa, and 1.5% in Lusaka. The infection rate in Kinshasa was similar to that reported by the National Program against HIV and STD in 2015 ^14^. However other reports show different rates. Taylor M. *et al.* analyzed seropositivity for syphilis during pregnancy among women screened at 40 ANC clinics in DRC in 2011. Women were screened with a treponemal test and those positive were confirmed with a non-treponemal test. The rate reported for clinics in Kinshasa was 2.2% ^9^. Other reports from West and Central African countries, including DRC reported rates of syphilis infection of 3.5% among pregnant women ^11^. Wijesooriya *et al.* ^3^ reported a 4.2% of infection using model based data, which was 2.4% after adjustment by the correction factor. Similarly, either the crude or the adjusted rate reported for Lusaka were lower than in previous reports. Makasa *et al.* ^15^ reported 2.3% prevalence of syphilis among pregnant women in Lusaka using Demographic and Health Surveys. And Strasser *et al.* ^16^ reported 3.5% positive rates in a sample of women screened during ANC. Both studies reported rates of confirmed cases using two tests.

One potential explanation of the discrepancy is that our study observed what happened under routine ANC conditions. Providers and laboratory technicians were not trained to perform and read the screening tests for this baseline study. Thus, under-detection is a possible explanation ^17, 18^. The fact that among women with a recalled history of previous syphilis that were screened using a treponemal test, no women in Kinshasa and only 2/48 in Lusaka had positive screening results (when 60% of positive results should have been expected), makes this possibility more likely ^13^. We acknowledge that other factors like chance can be possible explanations in particular for the Kinshasa low figures. On the other hand, a lower incidence of HIV infection have been reported in adult population in DRC, than in other Sub-Saharan countries including Zambia ^19^. It would then be possible that other infections like syphilis follow a similar pattern.

For women who tested positive, 10.0% of them in Kinshasa and 11.9% in Lusaka received treatment with benzathine penicillin in the first ANC. These figures are lower than other reports, which showed 63.6% of treatment at the first ANC visit in Lusaka ^16^. Shortage of benzathine penicillin treatment has not been reported as a barrier for treatment at the first visit in the trial formative research study. Treatment at any time during pregnancy was 90.0% in Kinshasa and 72.5% in Lusaka suggesting that the barriers are more related to treatment immediately after diagnosis, than to the lack of supplies. Provider´s barriers such as lack of awareness that one dose of benzathine penicillin would prevent congenital syphilis, resistance to treating women immediately after screening without treating the sexual partner and lack of time to deal with difficult cases and concerns about adverse reaction to treat women following many hours of fasting were reported in Lusaka and Kinshasa ^10^.

## Conclusions and implications for research

Our results show that screening for syphilis in pregnancy is not universal even when supplies are available. We also found large variations between countries, with higher rates of seroprevalence but lower screening and treatment rates in Zambia compared to the DRC. Further efforts will be needed to make supplies available and to understand the causes of the interruptions of the supply chains. Our ongoing trial will evaluate the impact of a behavioral intervention to change practices and increase screening and treatment rates when supplies are available.

## Data availability

The data supporting the findings reported in this study have been uploaded to OSF: https://osf.io/gyj4h/ DOI 10.17605/OSF.IO/GYJ4H

Data are available under the terms of Creative Commons Zero "No rights reserved" data waiver (CC0 1.0 Public domain dedication)

**Dataset 1. Baseline Paper Database.**

Women data collected throughout the study period.

**Dataset 2. Baseline Paper Clinics**

Characteristics of participating clinics

**Dataset 3. Baseline Paper Days of Attention Kinshasa**

Participating clinics in Kinshasa and days of prenatal care attention during working days during study period

**Dataset 4. Baseline Paper Days of Attention Lusaka**

Participating clinics in Lusaka and days of prenatal care attention during working days during study period

## Supplementary material

**Supplementary Table 1.** Description of attending days and screening for syphilis rate by clinic at Kinshasa (DOCX).

**Supplementary Table 2.** Description of attending days and screening for syphilis rate by clinic at Lusaka (DOCX).
