# Supplementary material for: Syphilis screening and treatment in pregnant women in Kinshasa, Democratic Republic of the Congo and in Lusaka, Zambia: a cross-sectional study
Source: Gates Open Res. 2017 Dec 8;1:13. [Version 1] doi: 10.12688/gatesopenres.12768.1 (PMC5764227; doi:10.12688/gatesopenres.12768.1)

**S1 Table 1.** Kinshasa- Description of attending days and screening for syphilis rate by clinic


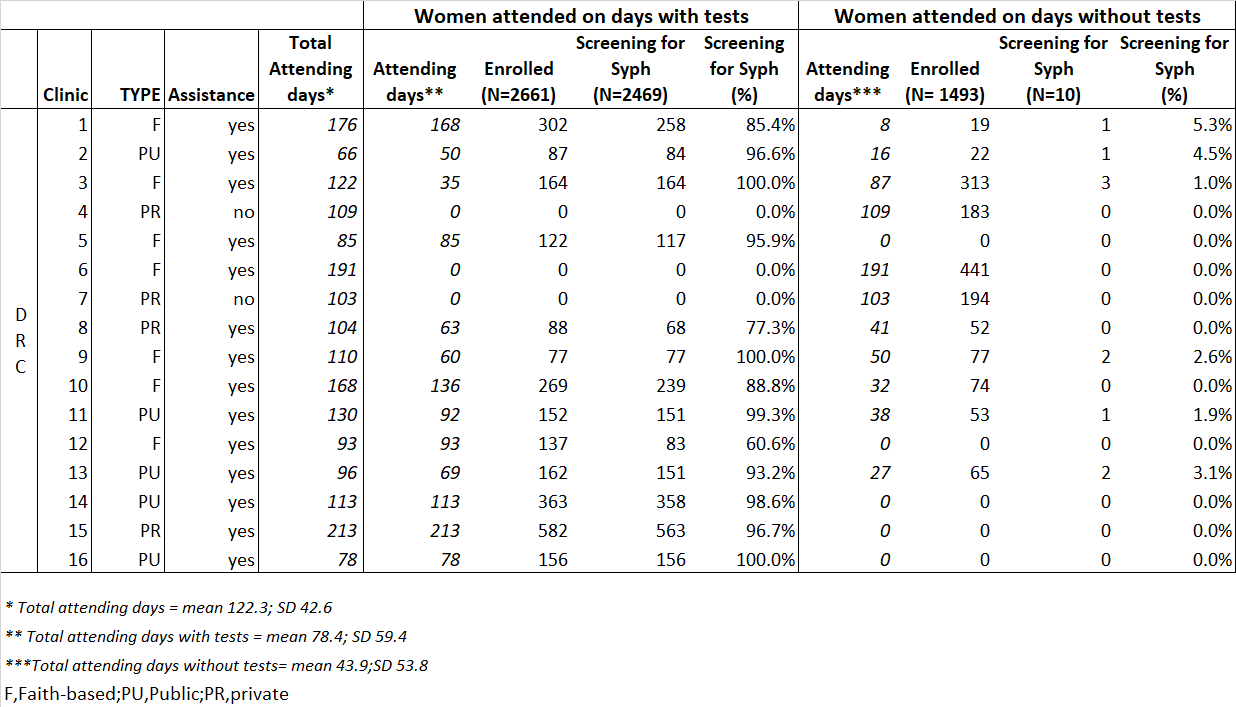


Table2. Lusaka- Description of attending days and screening for syphilis rate by clinic


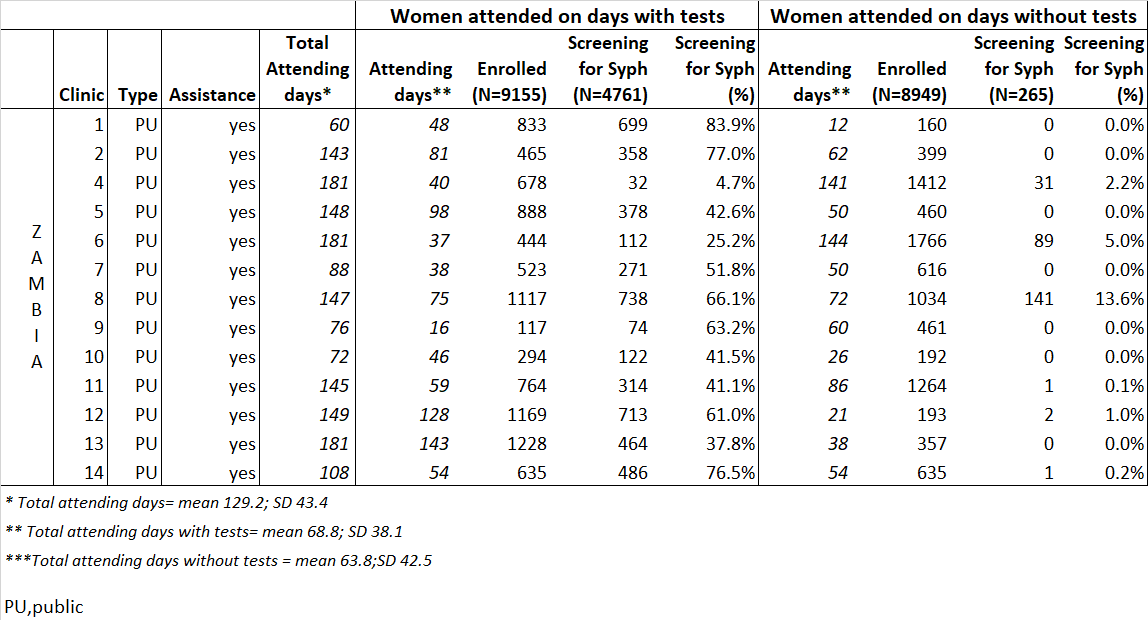

Supplement: Supplementary file 1 [file gatesopenres-1-13828-s0000.tgz › b42af3a5-6ce9-46f2-8bdc-0c80d6eb84ae.docx]
