# Supplementary figures and images for: Syphilis screening and treatment in pregnant women in Kinshasa, Democratic Republic of the Congo and in Lusaka, Zambia: a cross-sectional study
Source: Gates Open Res. 2017 Dec 8;1:13. [Version 1] doi: 10.12688/gatesopenres.12768.1 (PMC5764227; doi:10.12688/gatesopenres.12768.1)

**S2 Table 2.** Lusaka- Description of attending days and screening for syphilis rate by clinic


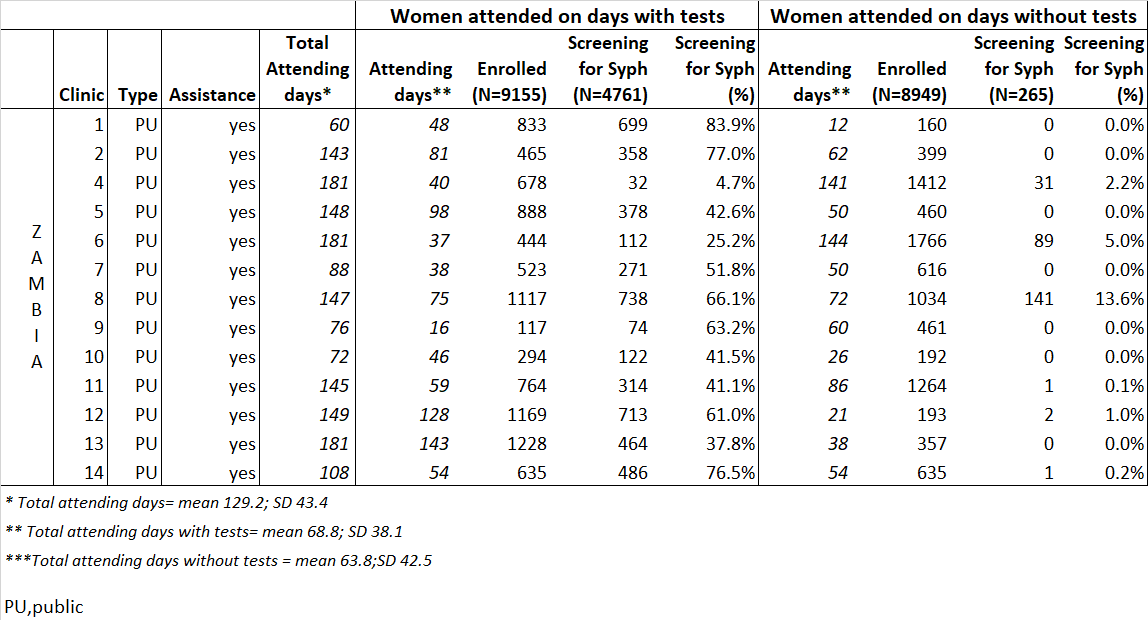

Supplement: Supplementary file 2 [file gatesopenres-1-13828-s0001.tgz › d34068b1-75a7-445e-99fd-532f882cac80.docx]
