# Supplementary material for: Syphilis screening and treatment in pregnant women in Kinshasa, Democratic Republic of the Congo and in Lusaka, Zambia: a cross-sectional study
Source: Gates Open Res. 2017 Dec 8;1:13. [Version 1] doi: 10.12688/gatesopenres.12768.1 (PMC5764227; doi:10.12688/gatesopenres.12768.1)
Supplement: Peer review history

# Peer review: Referee response for version 1

**DOI:** 10.21956/gatesopenres.13828.r26144

**Published:** 2018-01-09

**Authors:** Mary L. Kamb

**Affiliations:** 1 Division of STD Prevention, National Center for HIV/AIDS, Viral Hepatitis, STD and TB Prevention, Centers for Disease Control and Prevention (CDC), Atlanta, GA, USA

I thought this was a well written paper on a descriptive study of coverage of syphilis testing and treatment among pregnant women attending ANC clinics in Zambia and DRC. The study used baseline data from a facility-based randomized controlled trial that will evaluate a specific intervention. The information on coverage of testing and, especially, treatment is important because it relates to recommended antenatal intervention around prevention of mother-to-child transmission of syphilis (congenital syphilis), an important cause of perinatal morbidity and mortality in low income countries.

 I thought the background was well done, informative and provided appropriate citations. 

 The methods were clearly written and appropriate length. My only quibble was that I would have liked to have learned a little more about the intervention itself (just a sentence or two in the intro or methods, realizing that this is not the main focus of this paper). Figure 1 describing the clinics involved and the study enrollment was useful. 

 The results, indicating relatively low syphilis screening in both countries (particularly Zambia), and especially the very low treatment of positive women during the clinic visit (10-12%) were startling and important. I thought all tables provided were useful, and the data provided in the tables was well presented. A couple things that I would have liked to see reported in the text of the results, if not the tables, were (1) how representative were the women attending the chosen clinics with pregnant women (or even ANC attendees overall) in these two countries, and (2) were there differences in uptake of testing and treatment in different types of clinics (e.g., public, faith-based, other private).  

 I thought the discussion was particularly well organized and written, and especially appreciate seeing the discussion of study limitations. While the study did not specifically look at barriers, one important barrier cited in the literature that might be worth a mention under limitations is waiting time for women, i.e., if testing requires going to the lab and waiting for results, women may be unwilling to wait. It is interesting that testing was substantially lower in Zambia than DRC, perhaps due to use of RPR there (longer wait time). 

 Also in Discussion, the Montoya et al. citation is older, but probably still relevant in places where testing remains limited. Some more recent studies of rapid diagnostic tests (single syphilis RDTs and dual HIV/syphilis RDTs) indicate that these tests have moderate sensitivity against treponemal antigen (TP positivity) but relatively good sensitivity against higher titer TP, represented by TP positivity + non treponemal antigen positivity. This suggests that the problem of "over diagnosis and over treatment" using rapid treponemal tests in people with old, previously treated syphilis is probably less of an issue than initially feared. The current data suggest that most positive RDTs, especially in ANC women, probably need to be treated.

I have read this submission. I believe that I have an appropriate level of expertise to confirm that it is of an acceptable scientific standard.

# Peer review: Referee response for version 1

**DOI:** 10.21956/gatesopenres.13828.r26159

**Published:** 2017-12-28

**Authors:** Michael Marks

**Affiliations:** 1 Clinical Research Department, Faculty of Infectious & Tropical Diseases, London School of Hygiene & Tropical Medicine, London, UK

This is a well written paper describing baseline data from an ongoing cluster-RCT.

 I have only minor suggestions for improvement:

 1) In the abstract and throughout it would be preferable to refer to Mother-to-Child Transmission of syphilis as opposed to congenital syphilis as the former covers all manifestations including stillbirth whilst the latter technically only includes infection of infants born alive.

 2) It would be useful to understand what % of women in both settings do access ANC care vs presenting at labour. Potentially women who present late may be at higher risk of syphilis than women who attend early for ANC care.

 3) Did stock-outs of BenPen occur independent of stock-outs of screening kits? Might this explain the lower than anticipated same-day treatment rate for women who screened positive?

 4) I think it would be preferable to report timing of attendance by trimester rather than before/after 20 weeks as WHO recommends re-screening women in the third trimester. 

 5) Although I am aware this is baseline data from an ongoing trial I think it would still be useful to explain how the clinics were selected and to provide some sense of comparison to other non-trial clinics i.e how representative are the clinics in this study of all the ANC clinics in the two study sites?

I have read this submission. I believe that I have an appropriate level of expertise to confirm that it is of an acceptable scientific standard.
